# Monolayer Quantum-Dot Based Light-Sensor by a Photo-Electrochemical Mechanism

**DOI:** 10.3390/mi11090817

**Published:** 2020-08-28

**Authors:** Sitansu Sekhar Nanda, Minjik Kim, Sung Jong Yoo, Georgia C. Papaefthymiou, Dong Kee Yi

**Affiliations:** 1 Department of Chemistry, Myongji University, Yongin-si 17058, Korea; nandasitansusekhar@gmail.com (S.S.N.); kmjkim8323@gmail.com (M.K.); 2 Center for Hydrogen·Fuel Cell Research, Korea Institute of Science and Technology, Hwarang-ro, 14-gil, Seoul 02792, Korea; ysj@kist.re.kr; 3 KHU-KIST Department of Converging Science and Technology, Kyung Hee University, 26, Kyungheedae-ro, Dongdaemun-gu, Seoul 02447, Korea; 4 Department of Physics, Villanova University, Villanova, PA 19085, USA; gcp@villanova.edu

**Keywords:** CdSe, quantum dots, capacitor, semiconductor electrode, photo-sensor

## Abstract

Monolayer nanocrystal-based light sensors with cadmium-selenium thin film electrodes have been investigated using electrochemical cyclic voltammetry tests. An indium tin oxide electrode system, with a monolayer of homogeneously deposited cadmium-selenium quantum dots was proven to work as a photo-sensor via an electrochemical cell mechanism; it was possible to tune current densities under light illumination. Electrochemical tests on a quantum dot capacitor, using different sized (red, yellow and green) cadmium-selenium quantum dots on indium tin oxide substrates, showed typical capacitive behavior of cyclic voltammetry curves in 2M H_2_SO_4_ aqueous solutions. This arrangement provides a beneficial effect in, both, charge separation and light sensory characteristics. Importantly, the photocurrent density depended on quantum yield rendering tunable photo-sensing properties.

## 1. Introduction

Since their discovery in the 1980′s, semiconductor nanocrystals, also known as Quantum Dots (QDs) [1,2,3,4,5,6,7,8,9,10,11,12,13,14,15,16], have gained much research attention. In particular, CdSe QDs are of interest due to their unique optical properties derived from quantum confinement and photoelectric conversion phenomena [17,18]. These optical and optoelectronic properties of QDs have already found various applications in solar cells, solid-state lighting, bioimaging, and nanoscale gas sensors [19,20,21,22,23,24,25,26].

In addition to applications of QDs utilizing exclusively their optical properties, there have been recent reports on novel approaches to use QDs as electrodes in electrochemical energy storage devices using photo-electrochemical systems [27]. For the first time, Bae et al. employed CdSe QDs synthesized by a hot injection method to fabricate high efficiency supercapacitors [27]. Interestingly, this approach demonstrated that QDs could be used to produce electrical signals in response to incident light in photo-electrochemical systems. However, the photoelectric response property of QDs in photo-electrochemical systems has not been studied quantitatively, yet.

Quantum dot solution-processed photo detectors were studied for the first time by Sargent et al. [28]. This method affords low cost, one-step device fabrication with comparable performance to epitaxially grown high cost devices. However, the quantum dots had to be deposited at a thickness of hundreds of nanometers between Au electrodes in order to achieve working functionality. Considering the high cost of quantum dots, the requirement of a thick-layer coating can nullify the economic gain of adopting the one-step solution process manufacturing. Therefore, the development of a thin layer or monolayer quantum dots coating process is very desirable in terms of cost efficiency.

Herein, we report on a one-step fabrication, solution-processed monolayer-nanocrystal-based-light sensors composed of CdSe QDs deposited on Indium Tin Oxide (ITO) electrodes. To the best of our knowledge, our work is the first report on photoelectrochemical light sensors using monolayered QDs. Previously published QD-based photosensors adopted a multilayer approach incorporating a ~800 nm thick QD layer for light reception and conventional photocurrent generation through electron hopping between QDs and transfer to Au electrodes, which required close packing of quantum dots [28]. In contrast, our method employs drop casting of surfactant coated, well dispersed QDs at monolayer thickness. Instead of electron hopping, photocurrent generation in our system is based on redox-reaction mechanisms between QDs and the ITO electrode in the electrolyte. Therefore, our method does not require the preparation of multilayer, close-packed QDs.

In addition, this work is the first to suggest that the reduction/oxidation electrode system in an electrolyte can have dual application as (a) photocurrent generator and (b) sensitive detector of small current density changes induced upon light illumination, as compared to current under dark conditions. We observed that CdSe semiconductor QD ITO electrodes exhibited significant enhancement of current densities in electrochemical cyclic voltammetry (CV) tests, when they were under visible light illumination. Such observations on novel electrochemical sensors could lead to various designs of next generation QD-based sensors, displays, high resolution imaging sensors, and solar energy harvesting devices.

## 2. Experimental Section

### 2.1. Synthesis of Quantum Dots

Semiconducting CdSe nanocrystals were prepared by procedures we have described previously [27]. In brief, cadmium oxide (0.4 mmol, sigma Aldrich (Aldrich, St. Louis, MO, USA), 99.9%) and octadecylphosphonic acid (0.8 mmol, Alfa (Alfa Aesar, Ward Hill, MA, USA)) were mixed with trioctylamine (19.75 mL, Alfa, 95%). For degassing, the reactant mixture was purged with Ar gas for 60 min, and heated to 300 °C with rapid stirring under Ar gas flow. The Se precursor was prepared from a Se powder (0.4 mmol, Alfa, 200 mesh 99.999%) and 2M trioctylphosphine (Alfa, 90%). The CdSe semiconducting nanocrystals were prepared by quick injection of 1 mL of Se precursor into the Cd-containing reaction mixture at 300 °C. Green QD was prepared within 2 min after reaching 300 °C, and within 3, and 5 min for yellow and red QDs, respectively. After, 5 min the reaction mixture was cooled to room temperature. By adding anhydrous ethanol to the final product, a quantum-dot precipitate was obtained, and free surface additives were washed away by both hexane and acetone, three times. The product was redisposed in cyclohexane (1 mg/mL) for further characterization and sensor application.

### 2.2. Characterization of Quantum Dots

Optical properties of the synthesized quantum dots were studied using UV-Vis spectroscopy (Varian Inc., Cary 5000, Varian, Palo Alto, California, CA, USA) and fluorescence spectroscopy (Varian Inc., Cary Eclipse). Ethanol-solubilized Rhodamine 6G (quantum yield; 94%) was used as reference dye for measuring quantum yield. [29] The surface morphology of the quantum dots was characterized by high resolution transmission electron microscopy (TEM, Tecnai G2 TF 30ST, FEI, Hillsboro, Oregon, USA) interfaced with energy-dispersive X-ray spectroscopy (EDX).

### 2.3. QD Electrochemical Sensor Fabrications and Measurements

Quantum dots were deposited on indium tin oxide (ITO) substrates (surface area, 2 cm^2^) by a drop casting method (20 µL QD solution, concentration ~10^7^ particles/µL), and dried at room temperature. By several tests, we came to know that the controlled concentration of “~10^7^ particles/µL” asked two times (or less than) drop-deposition of 10 µL QD solution onto 2 cm^2^ area of ITO for making a monolayered QD layer; more than 20 µL dropping and drying under current QD concentration caused a multilayered QD film. [30,31] All electrochemical measurements were performed with a three-electrode configuration. As-prepared quantum dots on ITOs were utilized as working electrodes. A solution of 2M H_2_SO_4_ (aq) was used as an electrolyte. The cyclic voltammetry curves were taken using PARSTAT 2273 potentiostat (Princeton applied Research) in the potential range of −0.6 V to 0.2 V with 0.02–0.12 Vs^−1^ scan rate. As a light illumination source, a Xenon lamp (Newport 6258, 300 W) was used. Weights of quantum dots (red, yellow and green) on ITO were measured using a balance (Adventurer, Ohaus). Typical weights were 0.005 mg for all three kinds of QDs (red, yellow and green QDs).

## 3. Results and Discussion

**Figure 1:**
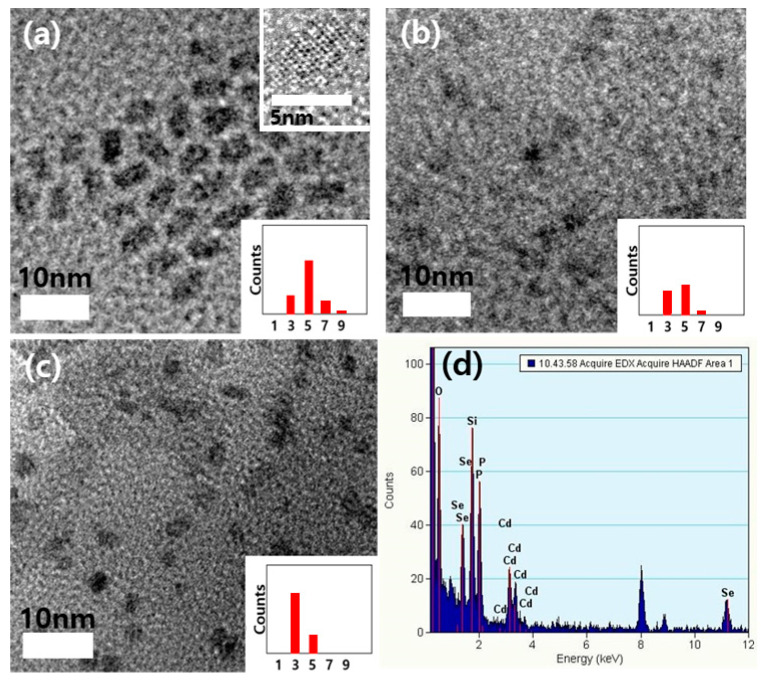
TEM micrographs of red, yellow and green QDs (**a**–**c**) and EDX analysis data (**d**) for red QDs; Inset for (**a**–**c**) shows the distribution of particle sizes.

Figure 1a–c show TEM micrographs of red, yellow and green CdSe QDs, respectively. The images show monodispersed QDs with average diameters of 6.5 ± 0.8 nm (red QD), 4.7 ± 0.6 nm (yellow QD) and 3.3 ± 0.4 nm (green QD). A histogram showing the particle size distribution is noted in the inset. A high-resolution TEM image for the red QD shows the lattice fringes (Figure 1a inset image). The EDX analysis data from the red QD shows that the QD consists of Cd and Se (Figure 1d). The EDX results from the other QDs (yellow and green QDs) also showed the same chemical contents of Cd and Se (data not shown).

**Figure 2:**
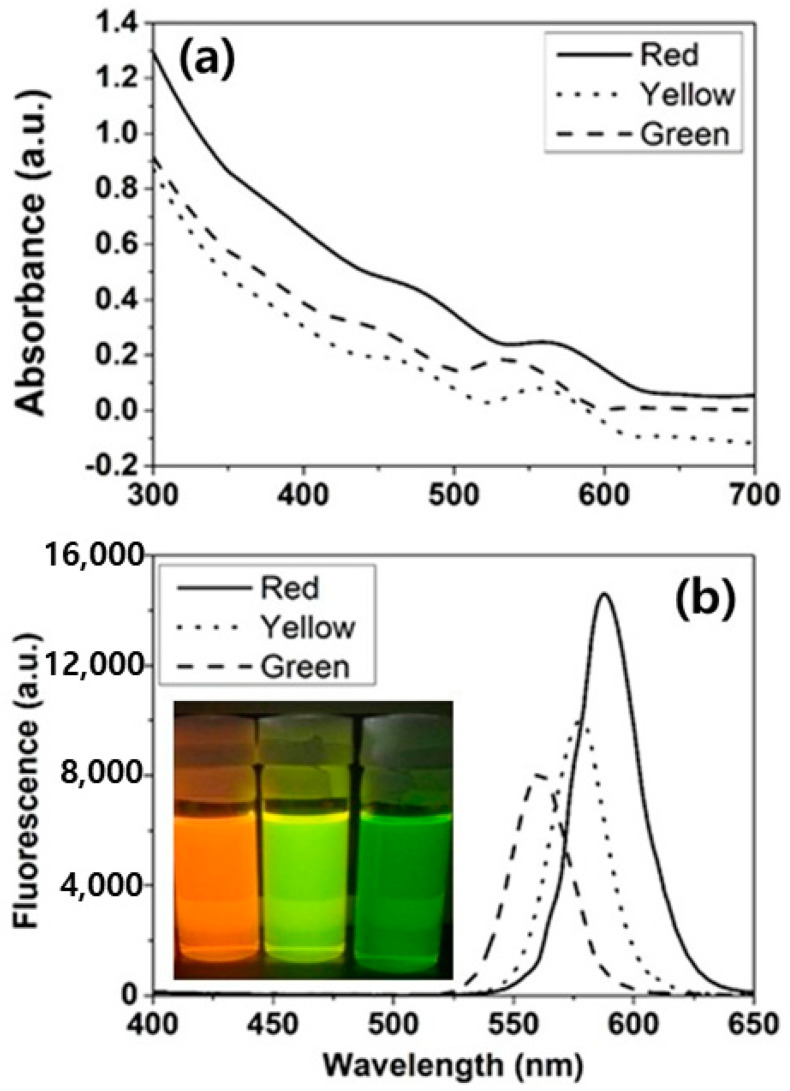
(**a**) UV-Vis absorption; and (**b**) photoluminescence spectra of red, yellow and green QDs. Inset of (**b**) shows the optical images under the UV lamp.

In Figure 2a, UV-vis absorption spectra of red, yellow and green Quantum Dots (QDs) are displayed. The excitation wavelength was 320 nm. The absorption peaks for the red, yellow and green QDs were 576 nm, 569 nm and 549 nm, respectively. Figure 2b displays photoluminescence spectra showing the peaks at 587.7 nm (red QDs), 578.1 nm (yellow QDs) and 561.3 nm (green QDs); inset in (b) shows the optical fluorescence observed for each QD-dispersion.

**Figure 3:**
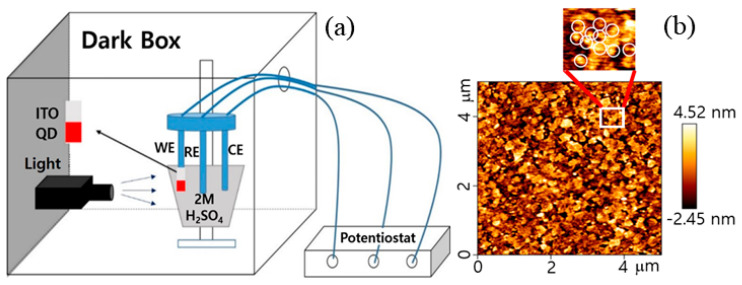
(**a**) A schematic showing the three-electrode configuration used for electrochemical measurements of QDs; (**b**) the arrays of QDs on the Indium Tin Oxide (ITO) electrode by AFM. Monolayered QDs are shown (one QD in a white circle, see the upper enlarged image).

A schematic of the three-electrode configuration used in the electrochemical measurements of the QDs is given in Figure 3a. The photo-electrochemical properties of the QDs were determined by CV investigations, which were carried out in the presence and absence of light illumination. The ITO substrates on which the QDs were deposited were employed as the working electrodes. Atomic Force Microscopy (AFM) was used to image the QD arrays, deposited on the ITO electrode. Figure 3b shows that the red QDs are well distributed along the ITO substrate as a monolayer. The image also indicates that individual QDs are well separated, not aggregated; shows the distribution of green and yellow QDs on the ITO. A Pt foil and Ag/AgCl in KCl were used as counter and reference electrodes, respectively.

**Figure 4:**
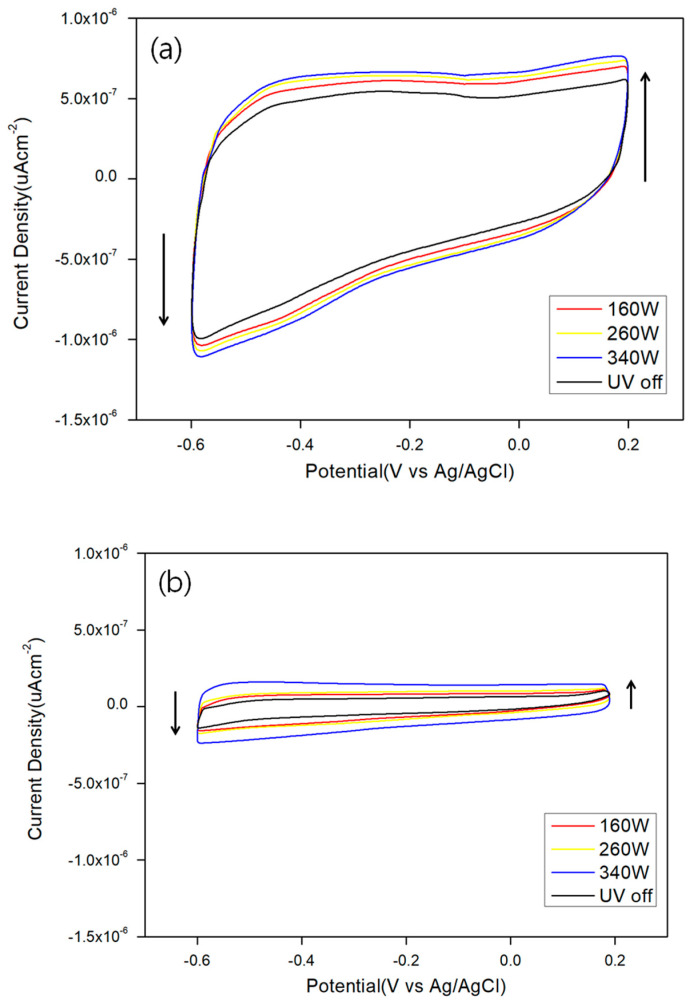

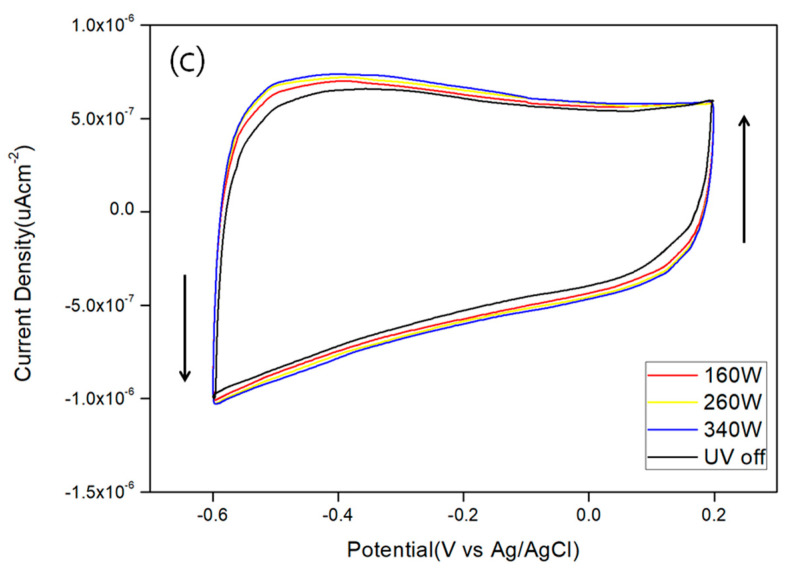
Cyclic voltammetry (CV) curves of red (**a**); yellow (**b**); and green (**c**) QDs under dark and light illumination with varying lamp wattages of 160, 260, and 340 W.

**Figure 5:**
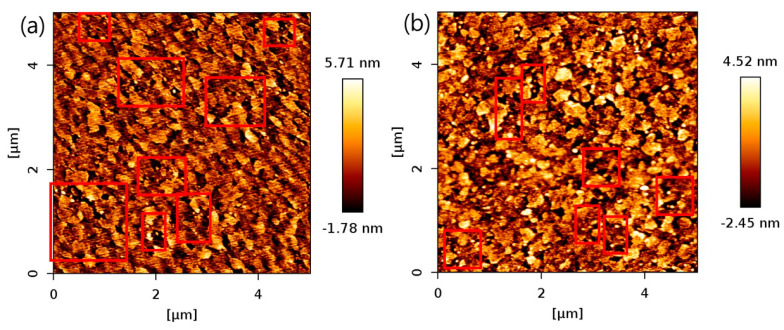
Atomic Force Microscopy (AFM) topographic data of QD arrays onto ITO surface for (**a**) yellow QDs; and (**b**) green QDs. The high-contrast spots in the red open boxes exhibit QDs.

We find that the enhancement of current densities under illumination take place for all three kinds of QDs. Figure 4 shows the CV curves of red (Figure 4a), yellow (Figure 4b), and green (Figure 4c) QDs under dark and illumination condition with varying power of the illuminating lamp. Figure 4a shows a CV curve of red QDs before and after illumination with varying lamp power of 160, 260, and 340 W. The current density increases with increasing light intensity, illuminating the red QDs on ITO. Figure 4b,c show CV curves for the yellow and green QDs on ITO under the dark and light illumination condition, respectively. The scan rate was 0.06 Vs^−1^. The Xe lamp was operated at 340 W. Both CV curves, under dark and light illumination condition, exhibited nearly rectangular curves, suggesting electric double layer capacitor (EDLC)-like charging and discharging behaviors. Evidently, CV measurements of QDs on ITO resulted in much higher currents under light illumination than those under dark condition. At the potential of −0.5 V, the current density with the QDs under illumination was approximately 0.6 μAcm^−2^ when the electrode was being charged by the potentiostat. On the other hand, the current density without illumination was ~0.2 μAcm^−2^, at the same potential. The illumination condition resulted in 3-fold enhancement of current density compared to the dark condition. It is also clearly seen that the current densities under illumination are much higher at all potentials ranging from −0.6 to 0.2 V. In Figure 5a, AFM topographic data of yellow QD arrays onto ITO surface measured, its height profile was 5.71 nm. In Figure 5b, AFM topographic data of green QD arrays onto ITO surface measured, its height profile was 4.52 nm. This result indicates the well distribution of QDs on ITO surface with its dissimilar size of QDs.

The three QD samples investigated had the same chemical composition but different sizes, rendering original fluorescent properties, i.e., they emit or absorb light at different wavelengths. The quantum yield (QY) for each type of QD was measured, using Rhodamine 6G (QY 94%) as a reference dye, to be 8.31, 11.25, and 6.51% for red, yellow, and green QDs, respectively. This order in QY value with respect to particle size was not repeated in the observed CV measurements. The highest QY (yellow QD) did not lead to the highest current density. Table 1 shows the highest current density values obtained for the reduction process at each incident light power used, at the potential of −0.4 V. Red QDs exhibited the highest values, while the yellow QDs showed the lowest values. Higher QY means that a larger number of photons are emitted from the surface of QDs per received photons. That is, under identical conditions, higher QY values indicate higher electron-hole production upon exposure to light and consequently a higher number of excitons. Therefore, higher QY should imply higher current densities, not observed here.

Integral values of CV graphs (area within CV loops) for each condition do not exhibit a clear relationship with QY (Table 2). However, they do show a proportional relationship with respect to exposing light power. In contrast, Table 3 shows that the comparative ratios (compared to that of light-off condition) of the integral values are in the order of QY values; the highest ratio, 2.91, is available when the yellow QDs (highest QY) are exposed to highest energy light, and the lowest ratio, 1.08, was available when the green (lowest QY) are exposed to lowest energy light. In addition, we also find this order (yellow > red > green QD) for each light power conditions (160, 260, 340 W). These comparative ratios represent the number of excited electrons over the number of non-excited electrons, at each condition. Therefore, this ratio is an important parameter in the evaluation of the photocurrent sensor sensitivity. The relationship between the QY and comparative integral values of CV graphs can be further studied to develop more sensitive photosensor structure. Therefore, QD monolayer with higher QY can be applied for highly sensitive photo-sensing devices.

## 4. Conclusions

In this study, we have showed that ITO electrodes on which a homogeneous monolayer of QDs has been deposited can work as photo-sensors via an electrochemical cell mechanism. For similar amounts of CdSe deposited on the surface of the ITO electrodes, QDs varied in their photo-sensing properties in accordance to what is expected due to quantum size effects. We have also demonstrated that the increase of current density in the QD electrodes depended on the intensity of the light used to illuminate the surface. Moreover, the resulting photo-sensor properties exhibited a clear relationship with the corresponding optical quantum yield values, when three different sized QDs, with same chemistry, were compared. Our development of CdSe QD for applications as photo-sensors can be further extended to novel photo-energy storage devices.

## Figures and Tables

**Table 1:** The highest current density values for red QDs, yellow QDs, and green QDs at −0.4 V.

μAcm^−2^	160 W	260 W	340 W	Light off
Red	7.00 × 10^−7^	7.20 × 10^−7^	7.39 × 10^−7^	6.53 × 10^−7^
Yellow	7.83 × 10^−8^	9.14 × 10^−8^	1.58 × 10^−7^	5.01 × 10^−8^
Green	5.62 × 10^−7^	6.12 × 10^−7^	6.40 × 10^−7^	4.83 × 10^−7^

**Table 2:** Integral values of cyclic voltammetry (CV) graphs for red QDs, yellow QDs, and green QDs, respectively.

μA-V/cm^2^	Light Off	160 W	260 W	340 W
Red	7.60	8.65	9.10	9.48
Yellow	0.76	1.15	1.37	2.21
Green	8.57	9.26	9.57	9.78

**Table 3:** Integral value ratio relative to light-off condition for red, yellow, and green QDs, respectively.

Ratio	160 W/Light Off	260 W/Light Off	340 W/Light Off
Red	1.13	1.20	1.25
Yellow	1.51	1.80	2.91
Green	1.08	1.12	1.14
